# Association of Obesity and Prolonged Hospital Stay With Surgical Site Infections After Coronary Artery Bypass Grafting in Intermediate- to High-Risk Patients: Insights From a Single-Center Study

**DOI:** 10.7759/cureus.99013

**Published:** 2025-12-11

**Authors:** Paraskevi Kyriazi, Athina Patelarou, Theodoros A Katsoulas, Evridiki Patelarou, Loukia Alexopoulou- Prounia, John Kokotsakis, Samiotis Ilias, Eleni Dokoutsidou, Despoina Alamanou, Konstantinos Giakoumidakis

**Affiliations:** 1 Department of Nursing, School of Health Sciences, Hellenic Mediterranean University, Heraklion, GRC; 2 Department of Nursing, National and Kapodistrian University of Athens, Athens, GRC; 3 Department of Cardiothoracic Surgery, Evangelismos General Hospital, Athens, GRC; 4 Department of Surgery, General Hospital of Athens "Elpis", Athens, GRC; 5 Department of Nursing, University of West Attica, Athens, GRC; 6 Department of Cardiology, 417 Veteran Hospital of Athens, Athens, GRC

**Keywords:** coronary artery bypass grafting (cabg), length of hospital stay, obesity, risk factors, surgical site infections (ssis)

## Abstract

Coronary artery bypass grafting (CABG) is one of the most common surgical procedures, contributing to improved long-term survival and quality of life for patients with coronary artery disease (CAD). However, surgical site infections (SSIs) remain a serious complication, increasing mortality, morbidity, and hospital length of stay.

Background/objectives: The study aimed to determine the incidence of SSIs and to identify factors associated with their development in patients at intermediate- to high-risk of infection undergoing CABG. It also aimed to provide context-specific evidence to inform targeted preventive strategies.

Methods: The study included 51 patients (39 (76.5%) men, 12 (23.5%) women; mean age 67.2 ± eight years) who underwent CABG via median sternotomy. Patients were preoperatively stratified for SSI risk using the Brompton Harefield Infection Score (BHIS), and only those classified as intermediate or high risk were included. The occurrence of SSIs was evaluated postoperatively using the Additional treatment, Serous discharge, Erythema, Purulent exudate, Separation of deep tissues, Isolation of bacteria, and Stay as inpatient prolonged over 14 days (ASEPSIS) scoring system. Data were analyzed using SPSS Statistics version 26.0 (IBM Corp., Armonk, NY, USA).

Results: Infections were observed in 17 (33%) patients. The occurrence of SSIs was positively associated with prolonged hospital stay (ρ = 0.512, p < 0.001) and obesity (f = 0.348, p = 0.013). Age, gender, smoking, diabetes mellitus, and ejection fraction were not statistically significantly associated with infection occurrence.

Conclusions: Obesity and prolonged hospital stay were significantly associated with higher SSI rates in patients after CABG. The high incidence of SSIs highlights the urgent need for targeted interventions in patients at increased risk. Early identification and proactive management of patients at increased risk may help reduce infection rates, improving postoperative outcomes and patient quality of life. The small sample size of this study, the data collection from a single cardiothoracic surgery center, and the limited number of examined variables are limitations that indicate the need for further research.

## Introduction

Cardiovascular diseases (CVDs) emerge as the primary cause of mortality worldwide. In 2019, they were responsible for almost 17.9 million deaths, which equates to 32% of all deaths globally [1]. It is a chronic condition that reduces blood flow to the heart muscle. In 2016, over half a million people in the European Union lost their lives to coronary diseases. Those over 65 were the most affected, with men being more vulnerable than women. Death rates from these heart conditions have decreased in recent years [2]. Coronary artery bypass graft surgery (CABG) has been a standard treatment alternative for CAD since 1960 [3]. In Greece, 473.2 CABG procedures per million population were performed in 2022, ranking among the highest in Europe [4]. In the USA, almost 40,000 such surgeries are performed each year [5]. Surgical site infections (SSIs) are estimated to take place in around 8.6% of patients undergoing CABG in the UK [6].

A plethora of risk factors may affect the development of SSIs that occur in patients after CABG. An SSI is one of the most significant complications in surgical patients and is associated with many adverse outcomes. Monitoring SSIs is a promising strategy for reducing infection rates and a valuable tool for evaluating the impact of various infection prevention interventions [7,8]. Surgical site infections are highly associated with increased mortality rates, length of stay, readmission, and healthcare costs. The healthcare costs due to SSIs in the USA can reach up to US$900 million a year [9,10].

Surgical site infections develop following CABG surgery and can involve only the superficial layers of the incision or extend deeper into the tissues [11-17]. The most severe form of SSI after CABG is sternal wound infection (SWI), especially deep sternal wound infection (DSWI). A DSWI is a rare but serious postoperative complication, linked to higher short-term and long-term mortality rates [18]. During CABG, the saphenous vein is commonly harvested from the lower limb for use as a graft. Complications in wound healing at the saphenous vein harvest site are much more common, with SSI rates reported between 2% and 20% [19,21]. Leg wound infections at donor sites are responsible for over 70% of severe infection cases following cardiac surgery [22]. An important proportion of SSIs are diagnosed only after hospital discharge. That way, the actual burden of infection may be underestimated, especially in studies that focus exclusively on data that are collected only when patients are still in the hospital [23]. 

Many studies have looked into the risk factors linked to SSIs after CABG, paying special attention to DSWIs, which tend to be more serious and carry higher risks for patients. One of the risk factors that often shows up when it comes to DSWI after CABG is diabetes mellitus (especially in patients requiring insulin or with poor glycemic control measured as glycated hemoglobin, HbA1c). Obesity, defined as BMI ≥30 kg/m², also stands out as a major factor. Age and female sex have been linked to increased vulnerability, as well as chronic obstructive pulmonary disease (COPD) and chronic kidney disease. Furthermore, the use of bilateral internal mammary arteries, extended surgical duration, and perioperative blood transfusions are all associated with increased DSWI risk. A hospital stay longer than 24 hours before surgery, along with postoperative complications such as prolonged mechanical ventilation and the need for sternal re-exploration, have all been independently linked to DSWIs [24-27]. The risk factors for SSIs in general (sternotomy or at the leg incision site used for graft harvesting), following CABG, include clinical and surgical characteristics. Reduced left ventricular ejection fraction (LVEF) below 45%), peripheral vascular disease, prolonged surgery time (particularly surgeries that tend to last over five hours), as well as the need for re-exploration or multiple blood transfusions, also affect the SSI rates. Extended preoperative hospital stays, undergoing emergency surgery, and poor glycemic control (HbA1c level above 7.5%) can also increase the likelihood of developing an infection [28-32]. 

The present study aimed to investigate the incidence of infections in patients undergoing CABG and to identify factors associated with SSI occurrence. In this context, the study sought to examine potential associations between patient and perioperative characteristics and the occurrence of SSIs, rather than to infer any causal relationships. Although several international studies have examined similar issues, data from Greek cardiac surgery populations remain limited, particularly among intermediate- to high-risk patients. Addressing this gap, the present research provides context-specific evidence from a tertiary hospital setting, aiming to contribute to a more comprehensive understanding of SSI incidence and factors associated with infection risk. This study also attempts to provide updated data on infection rates, highlighting associations that should be considered when healthcare administrators and clinicians design targeted preventive interventions.

## Materials and methods

Study design and variables 

This study followed a prospective observational design. The dependent variable was the incidence of SSIs, which include infections that may occur at the sternotomy site or the leg incision site after CABG. So, SSIs may present as SSWI, DSWI, and saphenous vein harvest site infections. Independent variables included patient sociodemographic characteristics (age, biological sex, marital status, and level of education), comorbidities (diabetes and obesity), lifestyle factors (smoking status), and clinical and operative parameters, including hospital length of stay and LVEF. Obesity was defined as BMI ≥30 kg/m² for purposes of statistical analysis of SSI risk. For Brompton Harefield Infection Score (BHIS) risk stratification, BMI ≥ 35 kg/m² was used per the original scoring system [31].

Participants and sample 

The study was conducted in the cardiothoracic surgery department and ICU of a large tertiary general hospital in the Attica region of Greece. Eligibility for study participation was determined based on specific inclusion and exclusion criteria. To be eligible, participants had to be 18 years of age or older, scheduled to undergo CABG via median sternotomy, able to read and write in Greek, and classified as intermediate or high risk based on BHIS, a stratification tool for predicting risk of SSI after CABG [31]. Additionally, written informed consent was required. Exclusion criteria included recent substance or alcohol abuse, as well as active infection within two weeks before surgery. Patients with a preoperative hospital stay longer than two days, those undergoing combined surgical procedures involving the aorta or heart valves, or those scheduled for emergency or urgent CABG were also excluded. Further exclusion criteria involved resternotomy for bleeding control or cardiopulmonary resuscitation and incomplete sternal closure at the time of ICU admission. 

Based on the above criteria, a convenience sampling method was used to recruit participants who underwent isolated CABG between April 2024 and January 2025, resulting in a final sample of 51 patients. The sample size was determined by the number of eligible patients undergoing isolated CABG at our center during the study period. A total of 40 patients were excluded: five declined participation, 10 failed to complete their follow-up checks as planned, two died, and 23 were classified as low risk for SSI. These exclusions were applied prior to determining the final sample of 51 patients.

Data collection and instruments 

Patients scheduled to undergo CABG who met the inclusion criteria were approached. Demographic and medical data were collected from each patient's medical records (both paper and electronic) and via clinical assessment. Patients arrived at least 24 hours before the scheduled surgery. A demographic data form was completed, and written informed consent for participation in the study was obtained. Risk stratification for the development of SSIs after CABG was performed for each patient using the BHIS [31]. This tool helps predict the risk of SSIs after CABG. Developed in the UK in 2015 by Raja et al., it analyzes 41 variables and identifies five key risk factors: diabetes or HbA1c >7.5%, BMI ≥35 kg/m², female gender, urgent surgery, and LVEF below 45%. Points are assigned as follows: 1 point for diabetes or 3 points for HbA1c >7.5%; 2 points for BMI ≥35 kg/m²; 2 points for female gender; 2 points for urgent surgery; and 1 point for LVEF <45%. Scores are added to classify patients into low (0-1), intermediate (2-3), or high risk (≥4) for developing an SSI after CABG [31]. Permission to use the scoring system was obtained from its original developers. Stratification was conducted preoperatively, on the day before surgery. Based on the preoperative risk assessment, patients that identified as intermediate or high risk for developing SSIs were included in the study sample. 

The presence of an SSI was assessed daily, clinically, and by using the Additional treatment, Serous discharge, Erythema, Purulent exudate, Separation of deep tissues, Isolation of bacteria, and Stay as inpatient prolonged over 14 days (ASEPSIS) scoring method [33,34] on each surgical wound that was carried by the patient. The ASEPSIS is a tool used to assess surgical wound infections, developed in England in 1986 by Wilson et al. [33]. It evaluates wounds based on symptoms such as serous discharge, redness, pus, and wound breakdown after cardiac surgery. Additional points are assigned for factors including antibiotic treatment, pus drainage, surgical cleaning under anesthesia, bacterial isolation, and hospital stays longer than 14 days. The highest weekly score determines the infection severity: 0-10 indicates satisfactory healing, 11-20 disturbed healing, 21-30 mild infection, 31-40 moderate infection, and over 40 severe infection. Use of ASEPSIS was authorized by its creators and the team who translated and validated it for the Greek population [33,34].

The assessment was conducted by the research team, following standardized ASEPSIS scoring procedures. Although formal inter-rater reliability testing was not conducted, all assessors adhered to the same protocol. Assessors were aware of patient characteristics, which may introduce assessment bias. Future studies should consider blinded assessment and formal reliability checks. A formal evaluation of patients' wounds, using the ASEPSIS tool, was also performed at the time of discharge, confirming that no patients had documented SSIs at that point. A close follow-up for possible SSI development was conducted on postoperative day 14, which is in accordance with the scheduled suture removal. On postoperative day 30, patients returned to the hospital for wound reassessment. For the first three months, follow-up evaluations were conducted at hospital facilities every 15 days, and subsequently monthly for an additional three months, during which the ASEPSIS tool was consistently used. All patients were informed that they could contact their physician or the research investigator at any time if they experienced symptoms of an SSI (such as redness, drainage, etc.), whether they were in or out of the hospital. Each patient was monitored for an SSI for six months after CABG. 

Ethical considerations 

The study protocol was approved by the Scientific Council-Committee of Ethics & Deontology of Evangelismos General Hospital (approval no. 513) and the Research Ethics Committee of the Hellenic Mediterranean University, Greece (approval no. 7414/10.01.2024). Written informed consent was obtained from all participants before their inclusion. The study was conducted under the ethical standards of the Declaration of Helsinki. All personal information is confidential; participants had the right to withdraw from the study at any time without penalty. The study was also prospectively registered at ClinicalTrials.gov (no. NCT06586749). 

Statistical analysis 

The statistical analysis was performed using SPSS Statistics version 26.0 (IBM Corp., Armonk, NY, USA). Continuous variables were presented as mean ± standard deviation, while categorical variables were shown as counts (percentages). To examine the relationship between a continuous and a dichotomous variable, the Spearman correlation coefficient (ρ) was used. The phi coefficient (φ) was applied to assess the association between two dichotomous variables. For all tests, a p-value of less than 0.05 was considered statistically significant. 

## Results

The study sample consisted of 51 patients, i.e., 39 men (76.5%) and 12 women (23.5%), with a mean age of 67.2 ± 8 years. Of which, 46 (90.2%) patients were married or cohabiting, 25 (49%) had primary education, 24 (47.1%) had secondary education, and the remaining two (3.9%) had tertiary education. Eighteen (35.3%) of the participants were obese, 36 (70.6%) had diabetes, and 29 (56.9%) were smokers. Seven (13.7%) had an ejection fraction less than or equal to 45%. The mean length of hospital stay was 6 ± 1.1 days. 

The SSIs occurred in 17 of the 51 patients (33.3%). The presence of infection was found to be statistically significantly associated with days of hospitalization (ρ = 0.512, p < 0.001) and obesity (f = 0.348, p = 0.013). Specifically, more days of hospitalization and obesity were associated with higher rates of SSIs. Age (ρ = 0.014, p = 0.922), biological sex (f = 0.196, p = 0.161), smoking (f = 0.140, 0.318), diabetes mellitus (f < 0.001, p = 0.999), and ejection fraction (f = 0.040, 0.774) were not statistically associated with the occurrence of SSI. Table 1 presents the socio-demographic and clinical characteristics of the study participants, including variables such as sex, marital status, education, obesity, diabetes, smoking, and LVEF.

**Table 1:** Sociodemographic and clinical characteristics of the study’s sample LVEF: Left ventricular ejection fraction

Characteristics		Number (total n=51)	Percentage
Surgical wound infection	No	34	66.7%
	Yes	17	33.3%
Biological sex	Female	12	23.5%
	Male	39	76.5%
Marital status	Single/divorced/widowed	5	9.8%
	Married/in a civil partnership	46	90.2%
Higher level of education	Up to primary education	25	49.0%
	Secondary education	24	47.1%
	Higher education	2	3.9%
Obesity	No	33	64.7%
	Yes	18	35.3%
Diabetes	No	15	29.4%
	Yes	36	70.6%
Smoking	No	22	43.1%
	Yes	29	56.9%
LVEF <45%	No	44	86.3%
	Yes	7	13.7%

Table 2 summarizes the relationship between patient age and duration of hospitalization with the occurrence of SSIs. A significant correlation was found between longer hospital stay and SSI occurrence.

**Table 2:** Age and the number of days of hospitalization concerning the occurrence of SSIs SSIs: Surgical site infections

Parameter	Surgical wound infection				Test	p-value
	No		Yes			
	Mean	Standard Deviation	Mean	Standard Deviation		
Age	66.9	7.5	67.7	9.3	ρ(51) = 0.014	0.922
Total days of hospitalization	5.6	0.7	6.8	1.3	Ρ(51) = 0.512	<0.001

Table 3 presents the association between biological sex, obesity, diabetes, smoking, and LVEF <45% with the occurrence of SSIs. Only obesity showed a statistically significant association (p = 0.013).

**Table 3:** Biological sex, obesity, diabetes, and smoking concerning the occurrence of SSIs LVEF: Left ventricular ejection fraction, SSIs: Surgical site infections

Parameter		Surgical wound infection		Test	p-value
		No	Yes		
Biological sex	Female	6	6	f = 0.196	0.161
	Male	28	11		
Obesity	No	26	7	f = 0.348	0.013
	Yes	8	10		
Diabetes	No	10	5	f < 0.001	0.999
	Yes	24	12		
Smoking	No	13	9	f = 0.140	0.318
	Yes	21	8		
LVEF <45%	No	29	15	f = 0.040	0.774
	Yes	5	2		

## Discussion

In our study, we found that approximately one in three patients developed an SSI following CABG. Our study provides new insights into the incidence and factors associated with SSI occurrence in intermediate- to high-risk CABG patients, focusing specifically on obesity and length of hospital stay. Among the various parameters examined, obesity and prolonged hospital stay were the parameters most strongly associated with SSI occurrence. These results generally follow trends seen in previous studies, though some small differences in patient characteristics or effect size might exist. On the other hand, no clear associations were observed between SSIs and other factors, including diabetes, patient age, smoking status, or LVEF. 

While no significant associations were observed for variables such as sex, marital status, education, diabetes, smoking, or LVEF, possibly due to limited sample size, narrow variance, or potential type II error, it is notable that the overall SSI incidence in our cohort was high, with one in three patients developing an infection. This rate clearly exceeds the 2% to 10% typically reported in the literature for general CABG populations [35,36], as well as the 2% to 20% reported for saphenous vein harvesting site infections [19-21]. This elevated incidence is expected, given that our study focused exclusively on intermediate- and high-risk patients and once more underscores the clinical importance of monitoring and prevention.

Apart from the higher-risk profile of our patients, there are a couple of other factors that may help explain this difference in incidence. First, the variation in research methods across different studies is of primary importance. For example, some studies use active post-discharge surveillance to identify infections, whereas others rely exclusively on clinician documentation. Some studies do not include structured follow-up after hospital discharge, which likely leads to underestimation of SSI incidence. The above-listed issues could result in underreporting of SSIs. Reported rates may differ depending on how long patients are monitored, what counts as an SSI, and the specific diagnostic criteria used. In the study by Berg et al. [23], one significant finding was that in patients who underwent CABG, most SSIs were diagnosed after the patient was discharged from the hospital, suggesting that some SSIs may have been missed. Hospital protocols, antibiotic use, surgical methods, and wound care practices are major factors that can explain why the SSI rates vary among studies. 

A lot of research has looked into what might increase the chances of SSI. The most common parameters associated with SSI include obesity and diabetes mellitus, as well as perioperative and postoperative complications [35,37-40]. The influence of patients' relative body weight on both early and long-term outcomes following cardiac surgery has been a topic of ongoing debate. Obesity comes up as an important factor associated with higher SSI rates after CABG. Adipose tissue produces some cytokines that have effects on immune cells. That way, the body’s ability to fight infections can be impaired. A general trend of increasing risk of SSIs has been observed as BMI (kg/m²) increased from normal levels to morbid obesity across nearly all types of surgeries [41]. In terms of CABG, morbidly obese patients are generally at a higher risk for SSIs, with obesity being an undeniably major factor associated with SSI occurrence [42]. 

Also, as stated earlier, it is well-known that people with diabetes tend to develop infections after surgery [43]. Diabetics are more likely to get SWIs after CABG compared to non-diabetic patients [44]. While diabetes is often deemed a major factor on its own for SSIs [43], we didn’t find a significant association in this particular group of patients. This result was somewhat unexpected, considering what has been found by other researchers. Possible explanations include the relatively small sample size, the single-center design of our study, and the high proportion of diabetics within our cohort, reducing the variability needed to unveil differences. We also didn’t observe a clear association between other factors like age, smoking, or ejection fraction and SSIs. A possible explanation is the relatively limited sample size in our study, which may have made it harder to discover smaller differences. It might also be that our patient group was relatively homogenous in certain characteristics (for example, age), limiting the ability of these factors to emerge as significant. 

Another important finding of our study was the strong association between the incidence of SSIs and the length of patient hospitalization, which is likely bidirectional. Staying in the hospital longer, on its own, can increase a patient's exposure to hospital-acquired germs, which raises the chance of developing an infection. The longer someone stays, the more likely it is that bacteria will settle on their body, making post-surgical infections more likely. Prolonged preoperative hospitalization has been recognized as a significant risk factor for healthcare-associated infections (HAIs). In the study by Sulzgruber et al. [45], extended preoperative stays were associated with increased incidence of SSIs among cardiac surgery patients. Even so, there is not a lot of research that focuses specifically on how longer hospital stays affect SSI rates in CABG patients. This particular aspect needs to be looked at more closely. Understanding this link better could help us find ways to lower the risk of SSIs.

Despite the new data provided, our study has several limitations. The sample size was relatively small, primarily determined by the number of eligible patients undergoing CABG at our center during the study period. While a formal power analysis was not performed, this study was designed as a preliminary investigation to identify potential associations and inform future, larger studies. Also, since all the data came from just one hospital, the results might not reflect what happens in other settings, where treatment protocols and patient profiles can differ. We also examined only a limited number of risk factors, due to our small sample size, which means we might have missed other important contributors. This choice was also deliberate, since adding too many variables in such a small dataset could create misleading results. For this reason, we decided to focus only on the most widely recognized risk factors, such as obesity, diabetes, smoking, ejection fraction, and length of hospital stay.

The SSI assessment was done daily by the research team using the ASEPSIS score [33,34]. Assessors were aware of patient characteristics, and inter-rater reliability was not formally tested. We acknowledge this as a limitation and suggest future studies include blinded assessment and reliability checks. Finally, without a multivariate analysis, we couldn’t fully explore which factors were truly contributing to SSIs, since we weren’t able to adjust for potential confounders. Larger studies including patients from multiple hospitals, examining a broader range of factors such as variations in antibiotic prophylaxis, surgical duration, and specific wound-care protocols, and applying multivariate analyses would help provide a more complete and reliable understanding of the factors influencing SSIs.

## Conclusions

Our study highlights that prolonged hospital stay and obesity are factors associated with, rather than causally responsible for, the increased risk of SSIs in patients undergoing CABG. These findings underscore the importance of early risk stratification, close postoperative monitoring, and the implementation of targeted preventive strategies, especially for intermediate- to high-risk patient groups. Nurse-led interventions, strict wound care protocols, and enhanced surveillance may help reduce infection rates and improve patient outcomes. Despite the valuable insights provided, the results should be interpreted with caution due to the study’s limitations, including the small sample size, single-center design, and lack of multivariate analysis. Future multicenter studies with larger sample sizes, a broader range of studied variables, and the use of multivariate analysis are needed to validate these findings and to support the development of evidence-based infection prevention protocols for this patient population.
